# The effect of ruminative thoughts on sexuality in obese women

**DOI:** 10.12669/pjms.40.1.8625

**Published:** 2024

**Authors:** Hulya Guc, Hasan Turan Karatepe, Asena Ayca Ozdemir, Ibrahim Bashan

**Affiliations:** 1 Hulya Guc, M.D. Mersin Akdeniz Yenimahalle Family Health Center Mersin, Türkiye; 2 Hasan Turan Karatepe, M.D. Department of Psychiatry, Istanbul Medeniyet University, Faculty of Medicine, Istanbul, Türkiye; 3 Asena Ayca Ozdemir, PhD, Department of Medical Education, Mersin University, Faculty of Medicine, Mersin, Türkiye; 4 Ibrahim Bashan, M.D. Department of Medical Education, Mersin University, Faculty of Medicine, Mersin, Türkiye

**Keywords:** Ruminative thoughts, Obesity, Sexuality

## Abstract

**Objectives:**

Ruminative thoughts play a significant role in the pathogenesis of disorders such as anxiety and depression. This study was conducted to investigate the impact of ruminative thoughts on the sexual functions of obese and non-obese women.

**Methods:**

This case-control study included individuals sexually active women aged 18 and above, and under 46 years, who applied to the university hospital’s obesity clinic in 2021 and had not previously been diagnosed with the patient and/or their partner with organic and/or psychiatric diseases that could cause sexual dysfunction and/or being under treatment, as well as pregnancy. The participants consisted of pre-obese and obese individuals (n = 59), as well as non-obese individuals (n = 63). The Ruminative Thought Style Questionnaire, Female Sexual Function Index, and Arizona Sexual Experiences Scale were administered to the voluntary participants. Using univariate and multivariate statistical models, the effects of obesity and volatile thought styles on the sexual experiences of obese and non-obese women were evaluated.

**Results:**

The analyses conducted revealed that the scores of ruminative thoughts were not affected by obesity. After adjusting for age, it was observed that Arizona Sexual Experiences Scale scores were higher in pre-obese and obese women (p<0.05). In the multiple models created, Arizona Sexual Experiences Scale scores were negatively influenced by obesity, whereas Female Sexual Function Index scores were adversely affected by ruminative thought styles.

**Conclusions:**

In this study, while addressing sexual health for both preobese, obese, and non-obese women, the importance of considering predisposing psychological factors is emphasized. It emphasizes the importance of ruminative thoughts and obesity on sexual functioning in women. Psychological well-being and body image perception emerge as critical elements in this relationship.

## INTRODUCTION

Sexual health and functions constitute a significant aspect of individuals’ quality of life, while the factors influencing this domain are highly intricate.^1^ Women’s sexual functions are shaped by the complex interplay of physiological, psychological, and social factors.^2^,^3^Among these factors are body image, self-esteem, stress levels, and psychological state.^1^,^4^ Ruminative thoughts (RT), which play a role in the pathogenesis of psychological disorders like anxiety and depression and are believed to negatively impact sexual health, also have a noteworthy association with sexual health.^5^,^6^ The relationship between RT and sexual health emerges as an important research area, particularly in the context of obesity, where this association remains relatively underexplored.^7^,^8^

RT refer to specific thought that can have an impact on mental health and functionality. These thoughts often recur, are frequently uncontrollable, and typically focus on negative or worrisome content.^6^,^9^ For instance, situations where an individual constantly dwells on past mistakes or future negatives can serve as examples of RT. In the literature, these thoughts are commonly referred to as rumination or mental anxiety. Rumination signifies the cyclical contemplation of a specific issue or problem, while mental anxiety may reflect a more general and persistent state of anxiety.^5^,^10^

On the other hand, obesity, which has emerged as a global public health issue today, is associated with numerous biological and psychological problems.^11^,^12^ Obesity often has negative effects on individuals’ body image and self-esteem. Moreover, obesity has been linked to psychological stress, anxiety, and depression, factors that can also negatively impact sexual functions.^13^ The fight against obesity still suggests that the most effective approach is the regulation of dietary habits and incorporating exercise, which, in addition to its direct effects on preventing weight gain and reducing (Body Mass Index) BMI, indirectly implies potential positive impacts (such as addressing ruminative thoughts disorders, sexual functions, etc.).^14^

This study aimed to investigate the potential relationship between RT and women’s sexual functions. Specifically, the focus was on examining the differences in this relationship between obese and non-obese women, with the anticipation that it will provide valuable insights into the field of sexual health.

## METHODS

This case-control study included a total of 171 women, as per the inclusion criteria These criteria encompassed women diagnosed by a psychiatrist, according to DSM-5 criteria had not previously been diagnosed with the patient and/or their partner with organic and/or psychiatric diseases that could cause sexual dysfunction and/or being under treatment, as well as pregnancy. Participants who did not meet the inclusion criteria were excluded from the research.

### Ethical Approval:

This study has been approved by the Istanbul Medeniyet University Göztepe Training and Research Hospital Clinical Research Ethics Committee of the Ministry of Health (02.12.2020-0697). After ethics committee approval was obtained, people who applied to Department of Family Medicine at Istanbul Medeniyet University Göztepe Training and Research Hospital in 2021 were included in the study.

**Fig.1:**
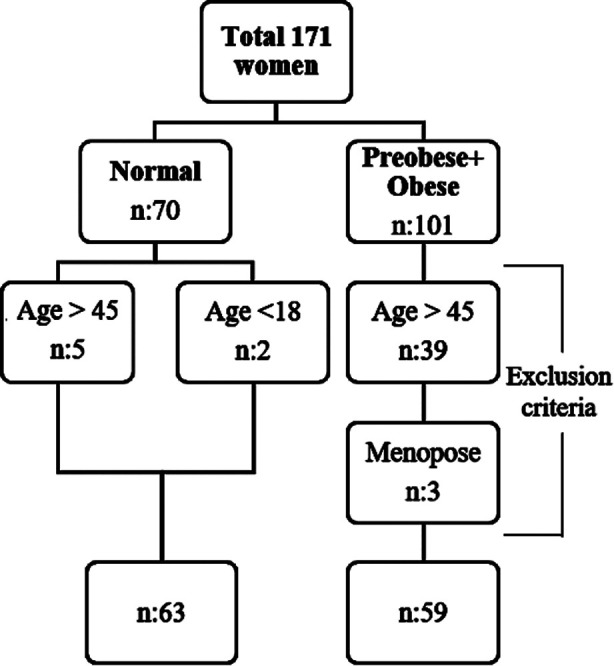
Flow Chart.

Out of the initial total, the number of women who did not meet the age criteria was 46, while the number of women aged 45 and under who had reached menopause was Three.. Consequently, a total of 122 women were included in the study (Fig.1).

Among the participants, 63 (51.6%) were in the normal BMI range (BMI <25), 21 (17.2%) were in the preobese (overweight) range (BMI ≥ 25, <30), and 38 (31.1%) were classified as obese (BMI> 30). The average BMI for women in the normal group was 21.67 ± 1.75 (min: 17.7- max: 24.9), while the average BMIs for women in the preobese and obese groups were 33.98 ± 7.36 (min: 25.5- max: 53).

### Measurement Tools:

### Ruminative Thought Style Questionnaire

The Ruminative Thought Style (RTS) is a ruminative and uncontrollable form of thinking. The scale, developed by Brinker and Dozois, consists of 20 items and is measured using a seven-point Likert scale.^15^ The Turkish adaptation of the scale was carried out by Karatepe.^16^ There is no specific cutoff point for scoring the scale. An increase in the total score obtained from the scale indicates an increase in RTS. The Turkish reliability coefficient of the scale is calculated as 0.91. In our study, it was found to be 0.95.

### Female Sexual Function Index

The Female Sexual Function Index (FSFI), a questionnaire consisting of 19 questions, was developed by Rosen and colleagues. Its Turkish validity and reliability were established by Aygin et al. This index is used by sexual health professionals to evaluate and comprehend women’s sexual function issues, and its primary purpose is to identify and guide treatment of sexual health-related problems by assessing various aspects of female sexual functions.^17^

The FSFI has six primary components: desire, arousal, lubrication, orgasm, satisfaction, and pain. Each component is evaluated with specific questions and a scoring system. By totaling the scores of these components, an overall score for sexual function can be calculated. Low scores may indicate potential issues with a woman’s sexual function, and this information can provide guidance for additional assessment and, if needed, intervention by sexual health specialists. The FSFI score is categorized as good if >30, moderate if 23-29, and poor if <23. The Turkish reliability coefficient of the scale is 0.79, whereas in our study, it was calculated as 0.98.

### Arizona Sexual Experiences Scale

The Arizona Sexual Experiences Scale (ASEX) is a self-report questionnaire developed to assess changes and disorders in sexual functioning.^19^ It comes in two separate forms for females and males, each consisting of five questions. Each question on the scale addresses sexual desire, psychological arousal, physiological arousal, capacity to reach orgasm, and post-orgasmic satisfaction. Each question is scored from one to six and the total score ranges from 5 to 30.

Individuals with a total score of 10 or below are considered to have a very low probability of having a sexual dysfunction based on psychiatric examination. A total score of 19 or higher, any item scored five or six, or three or more items scored four indicate sexual dysfunction and are highly associated with diagnosed sexual dysfunctions by clinicians. The Turkish version of the scale’s validity and reliability study was conducted by Soykan et al., and the reliability coefficient was reported as 0.88 and 0.92. In our study, the reliability coefficient was calculated as 0.87.^20^

### Statistical Analysis

Normality control of continuous variables was conducted using the Shapiro-Wilk test. In group comparisons where variables conformed to a normal distribution, the Independent Sample t-test was adopted, while the Mann Whitney U test was employed otherwise. Considering that age averages differed across the groups and could influence the outcome variable, we performed re-evaluation with the Analysis of Covariance. The multiple linear regression models were implemented to evaluate how the factors impact women’s sexual lives. The reliability of the scales was measured by Cronbach’s Alpha with a statistical significance level of 0.05 applied to all analyzes. The TIBCO Statistica® 13.5.0.17 program was used to analyze the data.

## RESULTS

The average age of the women included in the study was observed to be higher in the preobese and obese groups, and this difference was statistically significant (p<0.05). Although higher Ruminative Thought Style scores were observed in the preobese and obese groups, no statistically significant difference was found (p>0.05) (Table-I).

**Table-I:** Women’s Age and Ruminative Thinking Scores.

	Normal			Preobese+Obese			

	Mean±SD	Median (IQR)	Min-Max	Mean±SD	Median (IQR)	Min-Max	p
Age	32.52±5.35	31 (29-37)	22-45	37.47±5.96	38 (32-43)	24-45	<0.001
RTSQ	87.81±28.17	91 (67-110)	33-136	87.36±29.11	94 (67-109)	0-124	0.935

p: Mann Whitney U test, RTSQ: Ruminative Thought Style Questionnaire.

The evaluation of women’s sexual life according to obesity is given in Table-II. In the initial comparison of the groups, it was observed that the FSFI score was lower in preobese and obese women, indicating that their sexual functions were comparatively diminished. Both groups could be categorized as having a moderate level of sexual function (FSFI 23-29).

**Table-II:** Assessing the impact of obesity on women’s sexual activity.

	Normal			Preobese+Obese				

	Mean±SD	Median (IQR)	Min-Max	Mean±SD	Median (IQR)	Min-Max	p	p_adj_
FSFI	24.83±8.52	27.8 (21.8-30.2)	1.8-34.4	21.31±9.59	24 (17.1-29.3)	1.2-34	0.014^a^	0.190
ASEX	14.21±4.30	14 (11-17)	5-27	16.75±5.75	17 (13-21)	6-30	0.007^b^	0.033

p^a^:Mann Whitney U test, p^b^:Independent Sample t test, p_adj_: Age adjusted comparing, FSFI: Female Sexual Function Index, ASEX: Arizona Sexual Experiences Scale.

However, even after age adjustment, no significant difference was found in terms of FSFI values between the groups (p> 0.05). A score above 10 on the ASEX test indicates potential sexual dysfunction, which was observed in both groups. However, women with a BMI in the preobese and obese range had significantly higher ASEX scores. Multiple regression models were used to study the factors that affect women’s sexual experiences. Table-III The findings reveal that being obese increases the ASEX score by 2.09 units. Age and rumination were not found to contribute to sexual dysfunction (p>0.05). For FSFI scores, obesity and increasing age were associated with a decrease, but no statistically significant difference was detected. RT, on the other hand, was observed to contribute to a decrease in sexual function, and an increase of one unit in the RTSQ score led to a decrease of 0.06 units in the FSFI score (p<0.05). The resulting regression models were found to be statistically significant, explaining 6.2% and 6.8% of the variation in sexual experiences based on the variables in the model.

**Table-III:** Factors that affect women’s sexual life.

Dependent Variable	ASEX			FSFI		
	B (95% C.I.)	t	p	B (95% C.I.)	t	p
(Constant)	8.97 (2.52-15.42)	2.75	0.007	39.09 (27.7-50.47)	6.8	<0.001
Obesity	2.09 (0.11-4.06)	2.09	0.038	-2.16 (-5.65-1.32)	-1.23	0.221
Age	0.09 (-0.07-0.26)	1.14	0.255	-0.28 (-0.57-0.01)	-1.93	0.056
RTSQ	0.02 (-0.01-0.06)	1.54	0.126	-0.06 (-0.12--0.01)	-2.06	0.042
	*R^2^: 0.062, F:3.674, p:0.014*			*R^2^: 0.068, F:3.926, p:0.010*		

p: Multiple Linear Regression, R^2^: Coefficient of determination, FSFI: Female Sexual Function Index, ASEX: Arizona Sexual Experiences Scale, RTSQ: Ruminative Thought Style Questionnaire

## DISCUSSION

RT (Repetitive Thinking) is considered as a transdiagnostic process involved in the development and perpetuation of emotional disorders that adversely affect sexual function, although the exact mechanism of its impact remains not fully elucidated. In a study conducted by Peixoto and colleagues, which aimed to examine the effect of negative repetitive thinking disorders on sexual functions in a total of 424 participants, including 270 women aged between 18 and 72, it is shown that women are more predisposed to RT and have greater difficulty disengaging from these types of thought processes. 21 Our study was conducted exclusively with women that also found this score to be high. In studies where Nappi and Heidari at al., assessed sexual behaviors during menopause, negative changes in sexual behavior patterns of women during the menopausal period were evaluated;^22^,^23^ therefore, female participants in the climacteric and menopausal period were not included in this study.

To comprehend the impact of RT on obese and non-obese women, which in the climacteric and menopausal period have not been included in this study, and it is considered to be the first study of its kind found in the literature. Although higher RTS scores were observed in the preobese and obese groups, no statistically significant difference was found when compared to non-obese individuals.

Age has a potent impact on relationship quality and sexual functioning. The psychological influence of aging after midlife is a significant subject in terms of sexuality, relevant for both women and men.^24^ According to our study, women’s sexual experiences are influenced by obesity, but the effect of obesity on sexual experiences is not influenced by age. However, unlike obesity, rumination does not share a similar interaction with age; instead, it is observed to have negative effects on sexual health.

## CONCLUSION

This study demonstrates that RT patterns could be a significant factor affecting the sexual health of obese women. In the future, further research in this field could help us in understanding sexual health issues related to obesity and developing effective interventions.

### Author’s Contribution:

**HG:** Conceived, designed, editing.

**HTK:** Did data collection.

**AAO:** Did statistical analysis.

**IB:** Did manuscript writing, review, and final approval of manuscript and responsible for the accuracy of the work.
